# Episodic Memory and Executive Function Are Differentially Affected by Retests but Similarly Affected by Age in a Longitudinal Study of Normally-Aging Older Adults

**DOI:** 10.3389/fnagi.2022.863942

**Published:** 2022-04-13

**Authors:** Elizabeth L. Glisky, Cindy B. Woolverton, Katelyn S. McVeigh, Matthew D. Grilli

**Affiliations:** ^1^Aging and Cognition Laboratory, Department of Psychology, University of Arizona, Tucson, AZ, United States; ^2^Human Memory Laboratory, Department of Psychology, University of Arizona, Tucson, AZ, United States

**Keywords:** aging, episodic memory, executive function, longitudinal, retests, practice effects

## Abstract

Episodic memory and executive function are two cognitive domains that have been studied extensively in older adults and have been shown to decline in normally-aging older individuals. However, one of the problems with characterizing cognitive changes in longitudinal studies has been separating effects attributable to normal aging from effects created by repeated testing or practice. In the present study, 166 people aged 65 and older were enrolled over several years and tested at least 3 times at variable intervals (*M* = 3.2 yrs). The cognitive measures were composite scores. Each composite was made up of five neuropsychological tests, previously identified through factor analysis. For one pair of composite scores, variance attributable to age was removed from each subtest through regression analyses before *z*-scores were computed, creating two age-corrected composites. A second pair of composites were not age-corrected. Using linear mixed-effects models, we first explored retest effects for each cognitive domain, independent of age, using the age-corrected composites. We then modeled aging effects using the age-uncorrected composites after subtracting out retest effects. Results indicated significant retest effects for memory but not for executive function, such that memory performance improved across the three testing sessions. When these practice effects were removed from the age-uncorrected data, effects of aging were evident for both executive and memory function with significant declines over time. We also explored several individual difference variables including sex, IQ, and age at the initial testing session and across time. Although sex and IQ affected performance on both cognitive factors at the initial test, neither was related to practice effects, although young-older adults tended to benefit from practice to a greater extent than old-older adults. In addition, people with higher IQs showed slower age-related declines in memory, but no advantages in executive function. These findings suggest that (a) aging affects both memory and executive function similarly, (b) higher IQ, possibly reflecting cognitive reserve, may slow age-related declines in memory, and (c) practice through repeated testing enhances performance in memory particularly in younger-older adults, and may therefore mask aging effects if not taken into account.

## Introduction

Longitudinal studies of cognitive function in older adults have a relatively recent history, with the bulk of the research appearing in the literature since 2000. Much of this work initially focused on memory and speed of processing, areas of cognition that showed clear age-related differences in cross-sectional studies. More recent longitudinal studies have included other cognitive domains including executive function (e.g., Gross et al., 2015; Hassenstab et al., 2015), but in most studies, little has been said concerning what specific cognitive processes within a domain might be implicated in changes over time. In addition, studies have begun relating age-related cognitive changes to corresponding brain changes (e.g., Kramer et al., 2007; Persson et al., 2014; Armstrong et al., 2020; Gavett et al., 2021). Results of these longitudinal studies, however, have not been entirely consistent with respect to the cognitive domains most affected by age, the rate of decline over time, and the variables that might moderate change. Several researchers (e.g., Rabbitt et al., 2001; Ferrer et al., 2004, 2005; Rönnlund et al., 2005; Wilson et al., 2006; Salthouse, 2010) have also acknowledged that repeated testing can influence and thereby mask age-related changes, and have proposed different ways of accounting for and eliminating such effects. Although practice effects are usually greatest after short intervals, some studies have reported effects even 5-6 years following initial testing (e.g., Elman et al., 2018). It has also been suggested that practice effects themselves might reveal important individual differences in the cognitive functioning of older people (e.g., Machulda et al., 2013; Hassenstab et al., 2015).

For the present longitudinal study, we looked at composite measures of episodic memory and executive function in a sample of normally-aging older adults. Tests comprising each cognitive domain were chosen to reflect a common process, and the makeup of the composites was derived through factor analyses. We incorporated a novel way to separate aging and practice effects, and explored the impact of several individual difference variables on both retesting and aging.

We first began gathering neuropsychological test data from older adults in 1992 in the context of studying source memory. At that time, some studies had shown that on occasions when amnesic patients with medial temporal lobe damage recalled a recently-presented fact, they could also recall its source —where they heard it or who told them (e.g., Schacter et al., 1984). On the other hand, patients with damage to the frontal lobes, who could readily recall the facts, often could not recall their source (e.g., Janowsky et al., 1989). The two kinds of memory thus appeared to depend on different brain regions—recently presented fact memory on medial temporal lobe structures, and source memory on frontal brain structures. Subsequent studies reporting source memory deficits in older adults, further suggested that these deficits might indicate declining frontal lobe function in older people (e.g., Craik et al., 1990), but findings were inconsistent.

To test this hypothesis in older adults (Glisky et al., 1995), we chose tests from our neuropsychological battery thought to depend on each brain region. Specifically, we selected tests of episodic memory that varied in stimulus properties (e.g., verbal, visual, facial), encoding processes (e.g., single items, pairs, narratives), and retrieval processes (i.e., free recall, cued recall, recognition), but shared processes involved in the fundamental retention or consolidation of information over time, processes dependent on the medial temporal lobes. On the other hand, tests of executive function, thought to depend primarily on the frontal lobes, were selected to reflect control processes that were not involved in episodic memory, but instead were thought to be similar to executive processes associated with working memory. This assumption was supported in a later study by McCabe et al. (2010), who reported a high correlation (0.96) between our executive function composite (minus one common test) and a composite measure made up of complex span tasks.

To verify that these tests were indeed measuring separate constructs, we conducted a series of factor analyses. Because we were interested in the differential contributions of neurocognitive processes that were independent of age, variance attributable to age was removed from each individual test through regression analyses, and the residual scores were then submitted to factor analysis. The initial principal components analysis revealed two independent and uncorrelated factors. Composite factor scores, representing the average of the component test *z*-scores (equally weighted), were then assigned to each individual. Two later confirmatory factor analyses on separate and larger groups of older adults confirmed the two-factor solution and several follow-up studies showed that the two factors were differentially associated with item and source memory in older adults (Glisky et al., 2001; Glisky and Kong, 2008).

Rather than re-calculate and re-assign z-scores for each study sample going forward, we created a standardized reference group based on 227 community-dwelling older adults, who received these same tests, between 1998 and 2004. The data from this group then provided the means, standard deviations, and age corrections for classifying all past and future participants with respect to their episodic memory and executive function. We also created a parallel set of scores without the age correction, for studies in which age was a variable of interest (e.g., Glisky and Kong, 2008). The reference group, aged 65-90 (*M* = 73.4), had a mean education level of 15.6 years, were in good health, were not depressed or taking anti-depressant medications, reported no previous psychiatric or neurological problems that might have affected cognitive function, and had a score ≥26(**M** = 28.9) on the Mini-Mental State Examination (MMSE; Folstein et al., 1975). As our experimental studies continued over time, several people who had participated in our previous studies returned, and were re-tested to ensure that their cognitive profiles were up-to-date. Although not our primary goal at the time, this enabled the collection of longitudinal data, which, after several years, has allowed us to look at longitudinal changes in episodic memory and executive function and to contribute to this special issue on the importance of cognitive practice effects in aging neuroscience.

There are many reasons why practice effects should be considered in longitudinal studies of aging, many or all of which we expect will be addressed in this special issue. Our interests lay specifically in documenting and understanding the processes involved in “normal” cognitive aging, but because of repeated testing of the same materials and/or procedures, this was not a straightforward matter. Practice effects could elevate performance, making it difficult to assess the actual extent of normal aging processes. Several questions about practice could, and have been asked, including (a) Do all cognitive functions benefit equally from practice, and (b) what individual difference factors might influence practice effects? These were questions that we hoped to address with our data. In addition, understanding variability in the effects of practice might not only provide a greater understanding of the normal aging processes and the cognitive functions most affected, it might also help us in future studies to identify those individuals who were not aging normally, and perhaps suggest intervention strategies.

On the basis of prior studies, we expected that our episodic memory factor would show improved performance across testing sessions, and declines with increasing age. The few studies that have included measures of executive function and working memory have been inconclusive with respect to both retest and aging effects, and so suggested no clear hypotheses with respect to the executive function factor.

## Materials and Methods

### Participants

The present study includes data for 166 older adults between the ages of 65 and 91, who completed at least three testing sessions, were recruited continuously over a period of 18 years, and were retested at varying intervals (*M* = 3.2 yrs, *SD* = 1.4). The recruitment of participants for initial testing was conducted through the distribution of fliers in the local community, advertisements in the local paper, and public talks to groups at senior centers. Although some individuals continued to return for further tests (e.g., 83 people had at least 4 tests), we will focus here on the first three test sessions for which we have complete data. To ensure that our sample continued to warrant the label “normally-aging older adults,” we retained the exclusion criteria that we used for our standard reference group (see above), and removed people from the longitudinal study if they failed to meet those criteria in any of their test sessions. Those whose composite scores for either of the cognitive domains fell to more than 2 SDs below the mean were also dropped from further participation. Of the 547 older people who completed initial neuropsychological testing between 1992 and 2010, 53% (*N* = 292) completed Time 2 testing, and 58% of those individuals completed the third session. People failed to continue for a variety of reasons. Most dropouts were attributable to lost contact, lost interest, or ongoing physical or medical limitations. Of the 255 people who dropped between Test 1 and Test 2, 99 failed to meet inclusion criteria; 14 of those had neuropsychological scores more than 2 SDs below the mean, and 3 had MMSE scores below 26. Fifteen people failed to meet inclusion criteria for Test 3, two of whom had low MMSE scores. Three people were subsequently excluded because of missing FSIQ scores. Overall, those who dropped out tended to be older and had lower cognitive scores. All older adults in the present study, 114 women and 52 men, continued to perform within normal limits throughout all test sessions. Their mean age at Test 1 was 71.7 years (*SD* = 4.8), mean education 16.0 years (*SD* = 2.5), and mean MMSE score 29.1 (*SD* = 1.0). All studies that contributed data to the present study and their corresponding consent forms were approved by the University of Arizona’s Human Subjects Protection Program. Written informed consent was obtained on each testing occasion.

### Cognitive Tests and Measures

The primary outcome measures were the composite z-scores representing performance on the two uncorrelated neurocognitive factors, each derived from five neuropsychological tests. Tests contributing to the executive function (EF) factor included the number of categories achieved on the Modified Wisconsin Card Sorting Test (Hart et al., 1988), the total number of words produced to the cues F, A, and S in a verbal fluency task (Spreen and Benton, 1977), Backward Digit Span and Mental Control from the Wechsler Memory Scale-R or III (Wechsler, 1987, 1997b), and Mental Arithmetic from the Wechsler Adult Intelligence Scale-R (Wechsler, 1981). Tests representing episodic memory function (MF) included Logical Memory I, Verbal Paired Associates 1 and Faces 1 all from Wechsler Memory Scale-R or III (Wechsler, 1987, 1997b), Visual Paired Associates II from Wechsler Memory Scale-R (Wechsler, 1987), and Long-Delay Cued Recall from the California Verbal Learning Test (Delis et al., 1987). Two ***z***-scores were assigned to each participant for each cognitive factor, one representing age-corrected performance and the other age-uncorrected performance. Participants also completed IQ tests, the full tests prior to 1999 (Wechsler, 1981, 1997a) and the abbreviated version thereafter (Wechsler, 1999). Table 1 shows that at baseline (Test 1), individuals in the present study were on average 1.7 years younger than the reference group and performed at a somewhat higher level on the cognitive tests.

**TABLE 1:** Mean (sd) age-corrected composite z-scores, age, and FSIQ for reference group and study sample.

	Reference Group *N* = 227	Study Sample *N* = 166		
		Test 1	Test 2	Test 3
*Age-corrected EF Factor*	−0.0006 (0.66)	0.13 (0.62)	0.15 (0.62)	0.07 (0.66)
*Age-Corrected MF Factor*	−0.006 (0.63)	0.18 (0.62)	0.36 (0.63)	0.46 (0.60)
*Age (yrs)*	73.4 (5.4)	71.7 (4.8)	75.0 (4.9)	78.1 (5.0)
*FSIQ*	122.7 (13.8)	124.1 (12.2)	124.3 (11.4)	125.3 (12.1)

### Data Analysis

#### Practice Effects

We used linear mixed effects models to examine the longitudinal relation between repeated testing (1, 2, and 3) and age-corrected EF and MF scores. As noted above, variance attributable to age had been removed from these scores, eliminating any effects of increasing age across tests. The models included test session (centered such that test session 1 was the intercept) as our predictor of practice effects. The coefficient for test session reflects the longitudinal effect of repeated testing for each additional test session. To examine individual differences in the rate of change associated with one more test session, we also included age at baseline, sex, and baseline FSIQ, and their interactions with test session. We centered baseline age at 72 years, which was the round number closest to the average baseline age of the cohort. FSIQ was centered at the round number closest to the average FSIQ at baseline for the sample, which was 124. We included random intercepts in these models. Because test sessions 2 and 3 did not occur at fixed time intervals, we also ran the models examining practice effects on age-corrected EF and MF scores including two additional predictors: years since baseline, and the interaction between years since baseline and test session. However, these predictors were not significant in either model, and model comparison indicated that including them did not significantly improve model fit. For parsimony, we therefore did not include them in the final models examining practice effects.

#### Aging Effects

We applied the same linear mixed effects modeling approach to evaluate the role of increasing age on practice-corrected EF and MF scores. In these models, the primary predictor was years since baseline or “time,” to capture the effects of aging. The coefficient for time reflects the longitudinal effect of one more year of age on the cognitive outcomes. Whereas age-corrected scores were used to examine practice effects, practice-corrected scores were used to examine age effects. To derive these practice-corrected scores, we calculated the absolute difference between the age-corrected z-scores at session 1 and 2, and session 2 and 3, and subtracted the relevant difference scores from the age-uncorrected *z*-scores at session 2 or 3. Conceptually, this approach assumes that the differences between the age-corrected z-scores in session 1 and 2, and 2 and 3, primarily reflects the effects of practice, which are then removed from the age-uncorrected z-scores, creating the practice-free *z*-scores. These models also included age at baseline, sex, and baseline FSIQ, and their interactions with time to determine whether they influenced the age-related decline. As before, random intercepts were included.

RStudio was used for statistical analyses and data visualization (R Core Team, 2019), including lme4 (Bates et al., 2015), lmertest (Kuznetsova et al., 2017) to calculate p values, and “ggplot2” for data visualization (Wickham, 2016).

## Results

### Practice Effects

**FIGURE 1:**
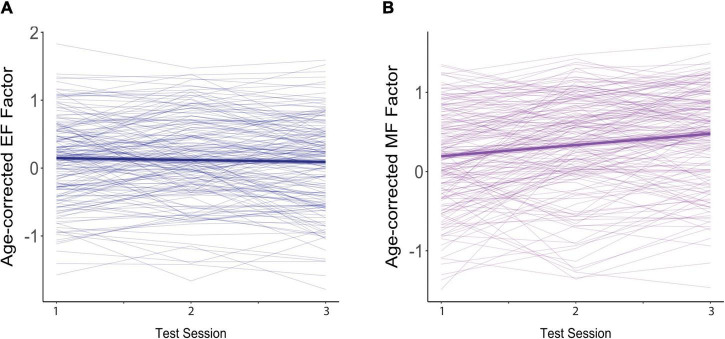
Effects of practice on age-corrected factor scores for **(A)** executive function (EF) and **(B)** memory function (MF). The dark blue (EF) and purple (MF) lines reflect the overall trend across test sessions. The colored ribbon around these lines is the 95% confidence interval. Each participant’s scores across the three test sessions are connected by a thin, light-colored line.

Mean *z*-scores for the two cognitive factors across the three test sessions are shown in Table 1. These composite measures are age-corrected such that increases in age over time cannot affect any boost in scores attributable to retesting. The data indicate little change in EF scores with repeated testing, but a substantial increase in performance on the MF tests. Individual data are shown in Figure 1. In this figure, each individual’s performance is represented by a thin blue (EF Factor) or purple (MF Factor) line, and the longitudinal effects of repeated testing from the linear mixed effects models described below are overlaid on these raw cognitive composite scores. Note that the interval between test sessions is variable across individuals (*M* = 3.2 yrs), and so does not represent continuous time.

**FIGURE 2:**
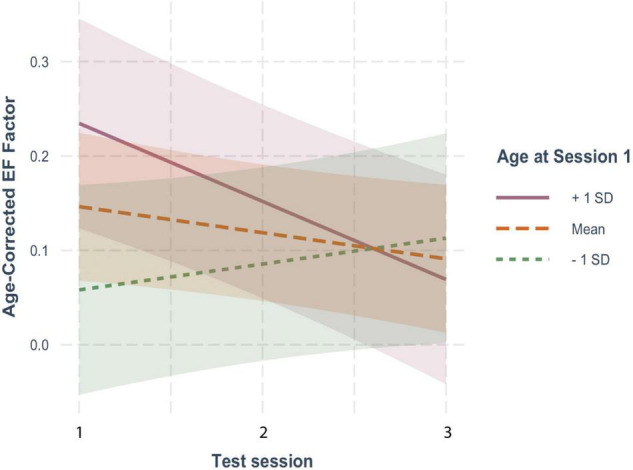
The moderating effect of baseline age on the rate of change in age-corrected EF scores across sessions. The solid line represents individuals who were on average 1 SD older than the mean age at baseline; the large-dashed line shows the performance of the mean age group, and the small-dashed line portrays those 1 SD younger than the mean age The colored ribbon around each line is the 95% confidence interval.

For EF (Figure 1A), age-corrected z-scores actually showed small but non-significant decreases with each repeated test (β = −0.027, *SE* = 0.019, *p* = 0.144), suggesting an absence of practice effects. This non-significant decline in EF scores was moderated by baseline age, as indicated by a significant interaction between baseline age and test session (β = −0.011, *SE* = 0.003, *p* < 0.001), but was not affected by FSIQ (β = 0.001, *SE* = 0.001, *p* = 0.306) or sex (β = −0.011, *SE* = 0.033, *p* = 0.729). As shown in Figure 2, although individuals older than the mean of 72 years on average (i.e., the old-older group (+1 *SD* = 5 yrs) showed a significant decline over test session (β = −0.079, *SE* = 0.025, *p* = 0.001), individuals at the mean or younger (i.e., the young-older group) on average showed neither a significant increase or decrease across test sessions (mean age: β = −0.024, *SE* = 0.019, *p* = 0.193; 1 *SD* below mean age: β = 0.031, *SE* = 0.024, *p* = 0.194). There was therefore no evidence of significant practice effects in EF in any age group.

**FIGURE 3:**
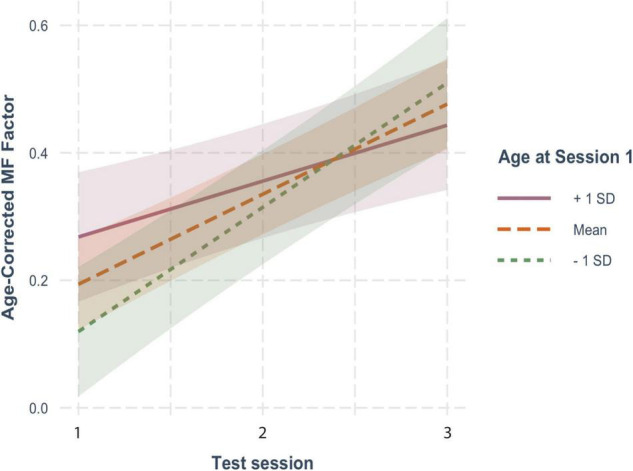
The moderating effect of baseline age on the rate of change in age-corrected MF scores across sessions. See Figure 2 for details.

On the other hand, for MF (Figure 1B), age-corrected z-scores showed clear and significant increases with each repeated test (β = 0.148, *SE* = 0.021, *p* < 0.001), reflecting practice effects. Here too, practice effects were significantly moderated by baseline age as reflected in the significant interaction with test session (β = −0.011, *SE* = 0.004, *p* = 0.002), but not by FSIQ (β = 0.002, *SE* = 0.001, *p* = 0.121) or sex (β = −0.030, *SE* = 0.038, *p* = 0.426). However, as shown in Figure 3, there were robust benefits of practice for MF scores regardless of baseline age (1 *SD* older than the mean: β = 0.097, *SE* = 0.028, *p* < 0.001; mean age: β = 0.151, *SE* = 0.021, *p* < 0.001; 1 *SD* younger than the mean: β = 0.204, *SE* = 0.027, *p* < 0.001). These practice effects, however, were smaller in those who were older on average at baseline, accounting for the interaction. Note also that preliminary analyses found no effect of time since baseline on practice effects, indicating that at long intervals (> 2 yrs), the number of years since the prior test did not predict practice effects.

Although only baseline age affected practice across testing sessions in either cognitive function, all of the individual difference variables contributed to cross-sectional differences in performance at baseline. For EF, there was a significant effect of baseline age (β = 0.018, *SE* = 0.008, *p* = 0.029), indicating that being older than 72 at baseline was associated with higher EF scores at the initial testing session. There was also a significant effect of FSIQ (β = 0.025, *SE* = 0.003, *p* < 0.001): Individuals with higher intelligence had higher baseline EF scores. Finally, there was a significant effect of sex, such that men had higher baseline EF scores than women (β = 0.203, *SE* = 0.087, *p* = 0.021).

For MF, there was also a significant cross-sectional effect of baseline age (β = 0.015, *SE* = 0.008, *p* = 0.044), indicating that being older than 72 at baseline was associated with higher baseline MF scores. There again was a significant effect of FSIQ (β = 0.020, *SE* = 0.003, *p* < 0.001): Individuals with higher intelligence had higher baseline MF scores. Finally, there was a significant effect of sex, such that women had higher baseline MF scores than men (β = −0.606, *SE* = 0.080, *p* < 0.001).

### Aging Effects

**FIGURE 4:**
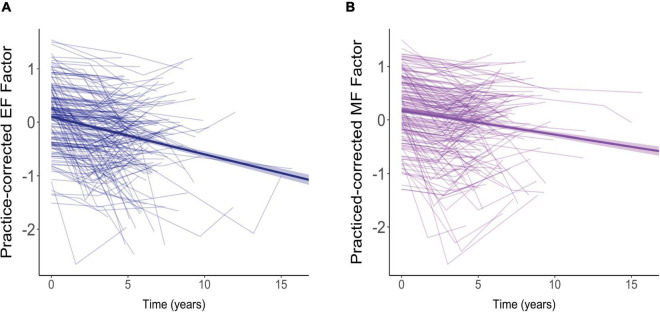
Effects of aging on practice-corrected factor scores for **(A)** executive function (EF) and **(B)** memory function (MF). Each participant’s scores across the three test sessions are connected by a thin, light-colored line. The dark blue (EF) and purple (MF) lines reflect the overall trend in factor scores with each additional year of age. The colored ribbon around these lines is the 95% confidence interval.

The effects of aging on our two cognitive factors are shown in Figure 4. For these analyses, we used practice-corrected EF and MF scores (regardless of whether there were significant effects of repeated testing) to ensure that age effects were not masked by practice effects. The longitudinal effects of time from the models below are overlaid on these raw cognitive composite scores.

For both EF (Figure 4A) and MF (Figure 4B), practice-corrected z-scores significantly decreased as time passed, indicating age-related cognitive decline in both cognitive domains (EF: β = −0.071, *SE* = 0.007, *p* < 0.001; MF: β = −0.041, *SE* = 0.007, *p* < 0.001).

**FIGURE 5:**
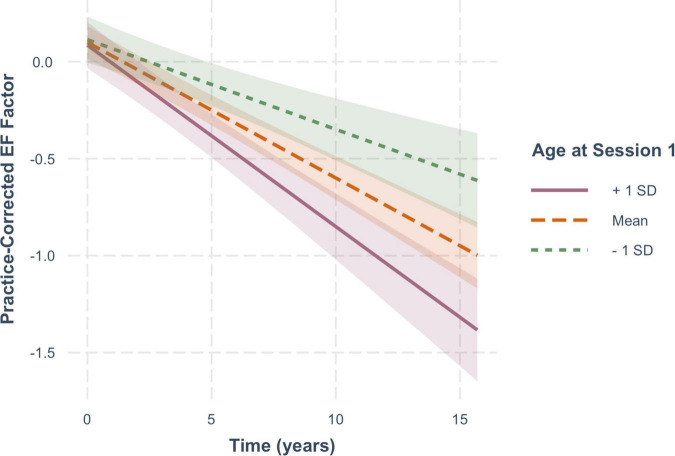
The moderating effect of baseline age on the rate of change in practice-corrected EF scores with each year of aging. See Figure 2 for details.

For EF scores, decline over time was significantly moderated by baseline age (*B* = −0.005, *SE* = 0.001, *p* < 0.001), but not by FSIQ (β = −0.0009, *SE* = 0.0005, *p* = 0.104) or sex (β = −0.00007, *SE* = 0.014, *p* = 0.996). As shown in Figure 5, practice-corrected EF scores significantly decreased over time regardless of baseline age (1 *SD* older than the mean: β = −0.094, *SE* = 0.010, *p* < 0.001; mean age: β = −0.07, *SE* = 0.007, *p* < 0.001; 1 *SD* younger than the mean: β = −0.046, *SE* = 0.010, *p* < 0.001), but the rate of decline was greater in individuals who were older on average at baseline, accounting for the interaction.

**FIGURE 6:**
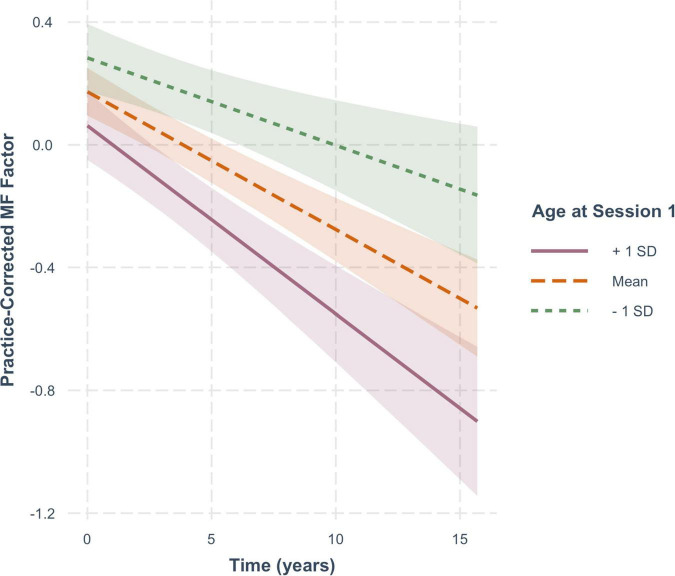
The moderating effect of baseline age on rate of change in practice-corrected MF scores with each year of aging. See Figure 2 for details.

**FIGURE 7:**
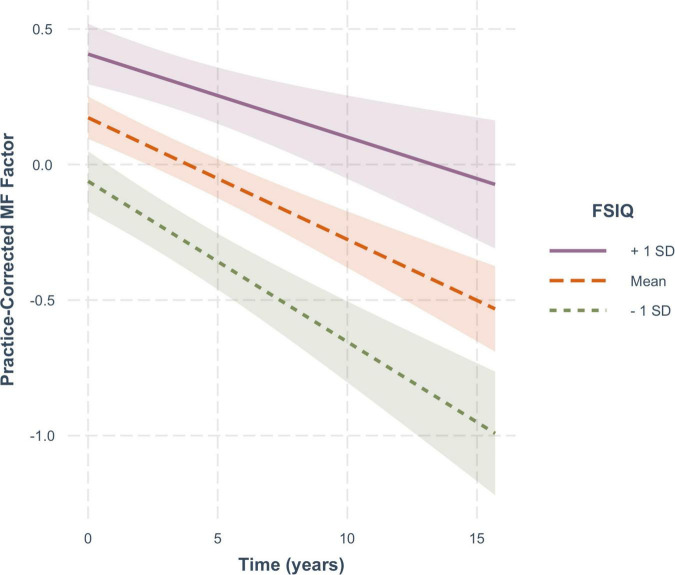
The moderating effect of full scale IQ (FSIQ) on the rate of change in practice-corrected MF scores with each year of aging. The solid line represents those who on average have FSIQs 1 SD above the mean, the large-dashed line shows the group at the mean, and the small-dashed line portrays those whose FSIQ scores were 1 SD below the mean. The colored ribbon around each line is the 95% confidence interval.

**FIGURE 8:**
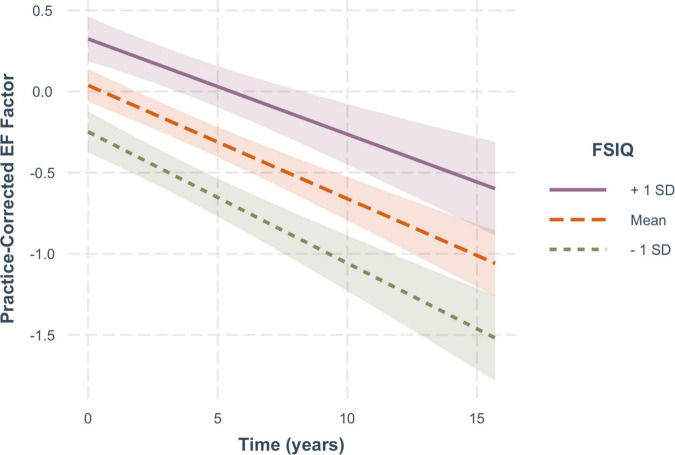
The non-significant moderating effect of FSIQ on the rate of change in practice-corrected EF scores with each year of aging. See Figure 7 for details.

For practice-corrected MF scores, decline over time was significantly moderated by baseline age (β = −0.003, *SE* = 0.001, *p* = 0.009), and also by FSIQ (β = 0.001, *SE* = 0.0005, *p* = 0.022), but not by sex (β = −0.016, *SE* = 0.013, *p* = 0.217). As shown in Figure 6, practice-corrected MF scores significantly decreased over time regardless of baseline age (1 *SD* older than the mean: β = −0.057, *SE* = 0.009, *p* < 0.001; mean age: β = −0.040, *SE* = 0.007, *p* < 0.001; 1 *SD* younger than the mean: β = −0.024, *SE* = 0.009, *p* = 0.007), but the rate of decline was greater in individuals who were older on average at baseline. Similarly, as shown in Figure 7, practice-corrected MF scores significantly decreased over time regardless of FSIQ (1 *SD* above the mean: β = −0.027, *SE* = 0.010, *p* = 0.007; mean FSIQ: β = −0.041, *SE* = 0.007, *p* < 0.001; 1 *SD* below the mean: β = −0.055, *SE* = 0.009, *p* < 0.001), but the rate of decline was slower in individuals who had higher FSIQs. Although FSIQ did not significantly moderate change in practice-corrected EF scores, this finding is shown in Figure 8 for comparison purposes.

Similar to the age-corrected scores, there were cross-sectional effects of FSIQ and sex at baseline. Higher FSIQ scores were associated with higher baseline scores on both cognitive measures (EF: β = 0.023, *SE* = 0.003, *p* < 0.001; MF: β = 0.019, *SE* = 0.003, *p* < 0.001). Men had higher baseline EF scores (β = 0.196, *SE* = 0.093, *p* = 0.037) and women had higher baseline MF scores (β = −0.615, *SE* = 0.087, *p* < 0.001). However, for these analyses of age-uncorrected scores, there was no significant effect of age on baseline EF scores (β = −0.003, *SE* = 0.008, *p* = 0.742), but there was an effect on MF scores (β = −0.023, *SE* = 0.008, *p* = 0.006), such that individuals who were older at baseline had lower baseline MF scores. This is consistent with the age correction being greater for MF than for EF scores.

## Discussion

In the present longitudinal study, we found that in a group of normally-aging older adults (65+), significant age-corrected retest effects (across three sessions in approximately six years) occurred in episodic memory but not in executive function. On the other hand, normal aging independent of practice effects, appeared to have similar effects on the two cognitive domains, resulting in significant declines in both cognitive functions. In general, young-older adults did better than old-older adults, showing greater practice effects and slower rates of decline with age. Interestingly, although full-scale IQ was associated with higher levels of performance at baseline for both cognitive factors, higher IQs did not enhance practice effects but were associated with a slower rate of age-related decline in memory; they did not significantly moderate the decline in executive function.

### Practice Effects

These results, as a whole, seem to make it clear that practice effects do not occur equally across all cognitive domains; that is, there is no general cognitive practice effect. Previous studies have reported similar findings. For example, Hassenstab et al. (2015) found practice effects in episodic memory, but not in several other cognitive domains including executive function (using tests similar to ours) (see also Wilson et al., 2006, However, Gross et al. (2015) did report practice effects in executive function, but with tests that were mostly non-overlapping with the ones used here. Elman et al. (2018), also using a different set of tests, found smaller practice effects in executive function than in episodic memory even in a younger group of adults (aged 50-60), but no practice effects in executive function when baseline cognitive ability was controlled. Together, these findings suggest that practice effects may be domain-specific, or possibly process-specific, occurring reliably in episodic memory but not in executive function, at least in the executive functions that were captured by our EF factor. Our findings also indicate that, when testing memory, one must account for practice effects because they can mask the effects of aging even at long delays (see also Rönnlund et al., 2005; Elman et al., 2018). As seen in Table 1, *z*-scores on the age-independent memory composite increase from 0.18 to 0.36 to 0.46 (*z*-scores) across the three tests, showing robust beneficial effects of repeated testing. If variance due to age had not been removed from those scores, those scores would have been 0.24, 0.29, and 0.28, and conclusions might have been that episodic memory seems to hold up well with normal aging. Nevertheless, our older people did show declines with age, once practice effects were removed.

So why did retesting improve memory but not executive function? For episodic memory, two possible answers to this question have generally been suggested (Goldberg et al., 2015): (a) People remember some of the actual stimuli from a first test and are therefore able to learn more, and (b) people develop memory strategies during the prior experience, which could later be employed to enhance memory further. Given the variety of memory tests that made up the composite measure, it seems unlikely that a common strategy or multiple strategies would have been learned during a single testing session. In the present study, memory continued to grow across two successive retests, suggesting that people were accessing the same memory representation and strengthening it on each occasion. We know that in the short term, repetition strengthens a memory trace. In the long term, retesting might enhance retrieval of a memory by presenting partial cues. New information might then be added to and strengthen the trace, which is then reconsolidated. In our sample, although the original memory traces may have weakened over time, they appeared still to be available and accessible when good cues were provided at retest. This explanation for retest effects fits well with the assumption that consolidation was the common memory process across the five tests that comprised the memory composite.

For executive function, although repetition might have allowed one to access the prior experience, it might not have helped one to perform the executive function tasks more efficiently. The tasks that comprised the EF factor in this sample all required attentional focus in the presence of interference, such as those involved in most working memory tasks. These kinds of tasks and processes lend themselves less well to the benefits of practice; gains tend to be short-term and task-specific, and require long hours of training (see Baddeley et al., 2015). Thus, it is not surprising that our EF factor did not improve across just two additional test sessions over several years.

Neither sex nor FSIQ influenced practice effects in either cognitive domain, suggesting that the practice effects that occurred in memory, may be at least partly automatic in normally-aging individuals. Whether you are a young-older person or amongst the oldest-old, intellectually gifted or less so, male or female, practice will enhance episodic memory. Our results did suggest, however, that improvements associated with retesting in the memory domain were smaller in the oldest, older adults. Interpretation of this finding, however, is not straightforward. It may reflect a decline in some automatic processes that are activated during retesting. For example, although cues from current tests may activate memory representations of prior sessions in older adults, the activation process might be slower or less complete at older ages. On the other hand, the representations themselves might be weaker in older adults, leading to a smaller increase over tests. It should be noted, however, that the baseline levels of performance at test session 1 differed across age groups, with the older adults having higher scores, and all age groups performing approximately equivalently by the third session. This suggests that ceiling effects might have reduced performance over time for those with higher levels of performance at baseline, in this case, the oldest group. The use of composite measures, however, limits ceiling effects and the composite scores did not appear to approach the ceiling. So overall, with respect to memory function, we conclude that normally-aging older adults of all ages show significant benefits of practice, although benefits may be smaller at oldest ages.

For executive function, there was a small but non-significant decrease in performance across test sessions indicating no effects of practice. Here, performance at baseline was also significantly higher for the old-older adults, and the scores also converged across age groups by the third test. Whereas the oldest group showed a significant decrease in performance across test sessions, the youngest group showed a non-significant increase. It is unclear why the oldest group’s performance would have declined across repeated tests. There may be another variable associated with re-testing that negatively impacts performance on our executive function tests. Alternatively, these results may reflect a regression to the mean. The bottom line, however, is that we did not find any significant benefits of retesting for executive function.

What might seem rather anomalous in these findings is that in both cognitive composites after age correction, the old-older adults, in general, were performing at a higher level at baseline than young-older adults. As noted earlier, however, individuals who dropped out of the study for various reasons or were removed for failing to meet inclusion criteria, tended to be older and had lower levels of cognitive performance. This resulted in a sample that was younger than the reference group on which their scores were based, leading to more below average composite scores amongst the young-older adults (i.e., a negative age correction) and higher composite scores amongst the oldest-old (i.e., a positive age correction). In the age-uncorrected data, the oldest adults showed the expected lower levels of performance, particularly in memory (see Figure 6, Time 0).

### Aging Effects

For these analyses, measures represent age-uncorrected performance levels. In Figure 5, one can see that there are no significant cross-sectional effects of age at baseline for executive function, but a significant effect of baseline age on memory function (Figure 6). This differential effect of age on the two cognitive composites accounts for the greater age-correction in memory than in executive function (see Table 1).

Aging effects, namely change in performance over time/years without any benefit from retests, are clearly evident in both cognitive domains. In addition, baseline age moderated both functions similarly, with the old-older people showing steeper declines over time than the young-older people. This finding is consistent with the notion that there might be a general aging-related factor common to the two domains (cf., Wilson et al., 2002; Salthouse, 2003).

At the same time, however, FSIQ, which was associated with baseline levels of performance for both cognitive functions, had no significant association with aging-related decline in executive function, but a significant moderating effect on memory function, such that those with higher IQs exhibited a slower decline in memory than those with lower IQs. This suggests an age-related process or function that differs across the two cognitive domains. Most longitudinal studies of aging have not included IQ as an individual difference variable although several have included education, with mixed results. We decided to include FSIQ, rather than education, primarily because of the different educational opportunities available to people across this wide age range, such that less education in our oldest old might not necessarily translate into lower intellectual function. We expected that IQ would incorporate not only acquired knowledge and skills gained in an educational context, but also a broader range of experiences and abilities acquired over a lifetime. In addition, education has often not shown any influence on age-related decline in memory (e.g., Zahodne et al., 2011; Wilson et al., 2019) or other cognitive functions (for review, see Seblova et al., 2020). In the aging literature generally, both education and IQ have been used as proxies for what has been called cognitive reserve (see Stern, 2007, 2009) and it is in this context that we will discuss the possible impact of IQ in the present study.

Here there are two related questions to be considered: Why is FSIQ associated with performance at baseline in both cognitive domains, and why does it moderate age-related decline only in memory? Reserve theory would suggest that baseline performance levels in both domains are related to brain reserve, which is established through the development of a structurally “better” brain (e.g., greater volume or connectivity) resulting from more varied life activities and experiences, and is reflected in the IQ measures. Brain reserve may benefit cognitive functions more broadly, as evidenced by the higher levels of performance at baseline in both executive and memory function for those with higher IQs. Although brain reserve is considered to be a relatively fixed entity at any one time, it also needs to be maintained over time presumably by continuing engagement in life’s activities (see Stern et al., 2020, 2022 for further elaboration). Cognitive reserve, however, refers to a more flexible and dynamic ability to adapt one’s cognitive processing in the light of declining brain networks. Thus, in the present study, high IQ at baseline may reflect greater brain reserve, which is supporting higher levels of memory and executive function at baseline, whereas the ability to moderate cognitive decline over time may reflect cognitive reserve, which may be domain- or process-specific. In memory, for example, older people tend to be more reliant on cues than younger people to retrieve episodic memories. Those adults with greater cognitive reserve may make more effective use of cues at retrieval, and therefore be more likely to reactivate a fading memory trace. On the other hand, the executive control processes associated with working memory, namely attentional focus under conditions of interference, may be less adaptable, and so less responsive to cognitive reserve. Note (see Figure 8) that there was a smaller but non-significant effect of FSIQ on age-related changes in executive function.

Overall, these results suggest that there may be both a common factor related to age-related declines in both cognitive functions, but also domain-specific factor(s) that might be differentially effective for different cognitive functions or processes.

### Implications

The present results indicate that both episodic memory and the executive functions associated with working memory decline with age. They also suggest that episodic memory may be more amenable to intervention than executive function in normally-aging older adults; practice improves memory and cognitive reserve helps to slow its decline. However, our sample included only people who were determined to be aging “normally,” and therefore does not speak to whether practice or cognitive reserve could be recruited to help those people with mild cognitive impairment or dementia. Prior studies that have included those with cognitive impairments are inconsistent in this respect with some studies showing improvements across retests (e.g., Gross et al., 2015) and others showing minimal or no effects (e.g., Hassenstab et al., 2015). In the present study of normally-aging older people, however, improvements in memory seemed to be available to even the oldest old, although perhaps to a somewhat lesser degree with increasing age.

From both a research and clinical perspective, the present findings have a number of implications. The results are based on a sample of people that are cognitively normal across all three testing sessions. They do not include people who have given any indication of underlying pathology that might affect cognitive function at any time over the years, although clearly, in the absence of any brain measures, we cannot rule that out. The sample, however, is relatively high-functioning with only a small number of individuals with IQs below 100. Although IQ is not reported in many studies, several have noted education levels of 16 years, comparable to our study (e.g., Wilson et al., 2006; Salthouse, 2010; Armstrong et al., 2020). Thus we do not think our sample is unique in that respect. It is, however, possible that although there was no effect of IQ on practice effects, those with still lower IQs might not show such benefits. Nevertheless, we think that the sample in this study is a good representation of normal cognitive aging in a community-based sample against which other comparable samples may be compared. We also feel confident in concluding that people who are aging normally should show practice effects on memory tests, but not necessarily on tests that require working memory or tax attentional resources. Failure to show retest effects on memory tests should therefore be considered a possible indicator of abnormal aging, which should be evaluated further.

Clinically, when assessing an older person on more than one occasion, especially in memory and even at long intervals, one needs to be aware that simply repeating the tests may confer some advantage and so scores may overestimate ability. One might want to choose different memory tests or materials at retest to offset, at least partly, the effects of practice, although if strategies were learned at initial testing, they might still provide some benefit. Accounting for practice effects may be particularly important for accurate diagnosis of mild cognitive impairment, particularly amnestic MCI. Eliminating the effects of retests may enable earlier diagnosis and intervention, which may prevent or slow the progression of the disease (see Elman et al., 2018; Sanderson-Cimino et al., 2022). Acknowledging possible effects of retesting might also be important in such things as clinical trials designed to evaluate the effects of a drug, for example (e.g., Goldberg et al., 2015). At the same time, if one is interested in interventions with real-world applications for normally-aging older adults, using a repeated testing procedure is a well-known strategy for enhancing memory over time (Roediger and Karpicke, 2006). Attempted retrieval of previously learned information has also been shown to improve memory and enhance learning of new information in people with memory impairments including those with Alzheimer’s disease (e.g., Pastötter and Bäuml, 2014). Retention intervals in these studies, however, are usually quite short (i.e., one month). There are thus both positive and negative effects of retesting: In longitudinal studies of aging, retesting may mask age-related declines in memory, leading to missed diagnoses of MCI, but in clinical interventions, retrieval practice may enhance memory in everyday life.

Finally, we would like to re-emphasize that the failure to find effects of practice or cognitive reserve in executive function very likely depends on the specific tests and processes. Executive function tasks rely on multiple processes, and although there may be a common factor across tests, there are clearly several different executive control processes grouped under the banner of executive function (e.g., Miyake et al., 2000; Glisky et al., 2021). Some of these may be modifiable by cognitive reserve or susceptible to practice, others may not. Looking at different types of executive functions longitudinally in an aging population would be an important future endeavor, which could identify more specifically the kinds of processes that are most amenable to modification. These findings also support the benefits of using composite measures made up of tests that might differ in many ways but share a common process. Being able to identify specific processes that are affected by aging, rather than focusing just at the domain level, could further enhance our understanding of aging and suggest interventions most likely to succeed.

### Strengths and Limitations

One of the major strengths of this study, as already noted, was the high probability that our sample included only older adults who were aging normally with respect to their cognitive function. This reduced the likelihood that any negative outcomes that we observed might be attributable to incipient pathology. At the same time, however, our sample was quite high functioning and may not be representative of the population in general. Second, as suggested and incorporated by many others, we used composite scores to reduce variability and error, but in our study (as in some others), the tests comprising the composites were chosen to reflect a common process determined through factor analysis. This allowed us to go beyond what many have said before about what cognitive domains are or are not affected by aging, and to begin identification of specific processes. Third, we believe that we have introduced a relatively novel way of separating practice and aging effects within an individual across repeated tests. Many studies have looked at practice effects across individuals, by comparing Time 1 performance in those who completed only Time 1 to those from the same cohort at Time 2, but this comparison is still between-persons and could be affected by other individual differences. Finally, we think that our results showing robust within-person practice effects in memory and no practice effects in our measure of executive function, make a strong case for concluding that not all cognitive functions show improvements with practice or retesting, and leaves room for many more studies to explore this issue at the level of processes. The findings with respect to aging also leave open the possibility that there may be (a) a common age-related factor that affects all cognitive processes, for example, global changes in the brain, (b) a common domain-related factor that affects all tests within a domain, or (c) process-specific factors within domains, dependent on more specific brain regions.

Limitations of our study include a relatively small sample size. In longitudinal studies that rely on community-based older adult volunteers who need to be available for several years, there are always many dropouts for a variety of reasons. In our case, to ensure that our sample continued to age normally, we also excluded people who had or developed psychiatric or neurological conditions that might affect cognitive function. Our sample size therefore limited to some degree the kinds of analyses that we could do and our ability to explore additional factors. Another limitation of our work is that we did not have any direct measures of brain integrity or function, which might support our cognitive findings. Although we suggested that the common factor among our memory tests most likely reflected consolidation dependent on medial temporal lobe regions, and our executive function tests depended on prefrontal brain regions associated with working memory, we could not determine that from our study, and certainly we could not be more specific. The recent advances in neuroimaging, however, which have begun to relate longitudinal changes in cognitive functions to corresponding changes in different brain regions (e.g., Persson et al., 2014; Armstrong et al., 2020; Gavett et al., 2021), will continue to lead to new ideas and discoveries that will add considerably to our growing understanding of both normal and pathological aging.

## Data Availability Statement

The raw data supporting the conclusions of this article will be made available by the authors, without undue reservation.

## Ethics Statement

The studies involving human participants were reviewed and approved by University of Arizona Human Subjects Protection Program. The participants provided their written informed consent to participate in this study.

## Author Contributions

EG: conceptualization, methodology, supervision, and writing – original draft, review and editing. CW: data curation, investigation, and project administration. KM: data curation and formal analysis. MG: formal analysis and supervision, methodology, visualization, and writing – original draft, review and editing. Based on contributor roles taxonomy (CRediT). All authors contributed to the article and approved the submitted version.

## Conflict of Interest

The authors declare that the research was conducted in the absence of any commercial or financial relationships that could be construed as a potential conflict of interest.

## Publisher’s Note

All claims expressed in this article are solely those of the authors and do not necessarily represent those of their affiliated organizations, or those of the publisher, the editors and the reviewers. Any product that may be evaluated in this article, or claim that may be made by its manufacturer, is not guaranteed or endorsed by the publisher.
